# Supplementary material for: Nanogallium-poly(L-lactide) Composites with Contact Antibacterial Action
Source: Pharmaceutics. 2024 Feb 4;16(2):228. doi: 10.3390/pharmaceutics16020228 (PMC10893416; doi:10.3390/pharmaceutics16020228)
Supplement: Supplementary file 1 [file pharmaceutics-16-00228-s001.zip › pharmaceutics-2828839-supplementary.pdf]

Nanogallium-poly(L-lactide) composites with contact antibacterial action – supplementary

Mario Kurtjak [1,*], Marjeta Maček Kržmanc [1], Matjaž Spreitzer [1], Marija Vukomanović [1]

[1] Jožef Stefan Institute, Jamova cesta 39, 1000 Ljubljana, Slovenia; marjeta.macek@ijs.si (M.M.K.); matjaz.spreitzer@ijs.si (M.S.); marija.vukomanovic@ijs.si (M.V.)

* Correspondence: mario.kurtjak@ijs.si

Supplementary materials and methods

Morphology of Ga/OA NPs was imaged in a Jeol JSM-7600F (JEOL, Tokio, Japan) scanning electron microscope operating at 5 kV. 20 μL of colloidal solution of Ga/OA NPs was dried on a piece of Si wafer, which was stuck on carbon tape and coated with 5 nm of carbon (using precision etching coating system (PECS) Model 682, Gatan, Pleasanton, CA, USA) before observation. Their size distribution was analysed using ImageJ software and normal distribution fitting in Python. TEM imaging of Ga/OA NPs was performed on a Jeol JEM-2100 transmission electron microscope operating at 200 kV. 20 μL of colloidal solution of Ga/OA NPs (in chloroform) was dried on a lacey carbon/Cu TEM grid for observation.

Diffuse reflectance infrared Fourier transform (DRIFT) spectrum of oleic acid (OA) annd Ga/OA NPs was measured in Perkin Elmer Spectrum 400 MIR spectrophotometer (Perkin Elmer, Waltham, Massachusetts, USA). Sample of OA or Ga/OA NPs was mixed with KBr by dropping a few μL of OA or Ga/OA colloidal solution onto KBr powder, drying in air and homogenizing by pestle in a mortar. For calculating the theoretical UV-VIS spectra of Ga NPs, a MATLAB program (The Metal Nanoparticle (MNP) simulator) created by Guido Goldoni was used. It is based on the MATLAB programs written by Christian Mätzler [1] using Mie scattering theory [2]. Dielectric function for liquid gallium was taken from ref. [3]. Refractive index of PLA was obtained from reference [4] and the average value between 220 and 280 nm (i.e., 1.52) was used in the calculation.

Supplementary images

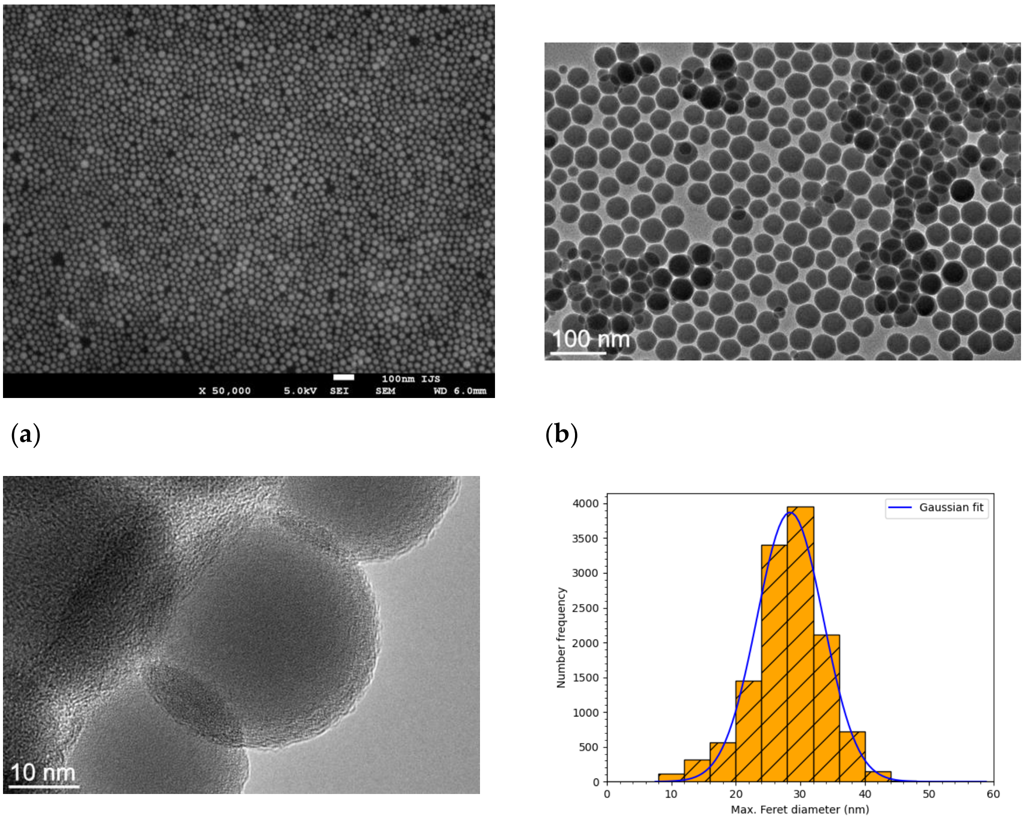

(a) (b)

(c) (d)

Figure S1. Colloidal Ga/OA nanoparticles: (**a**) morphology under SEM; (**b**) morphology under TEM; (**c**) detailed morphology by TEM; (**d**) size distribution based on SEM images.

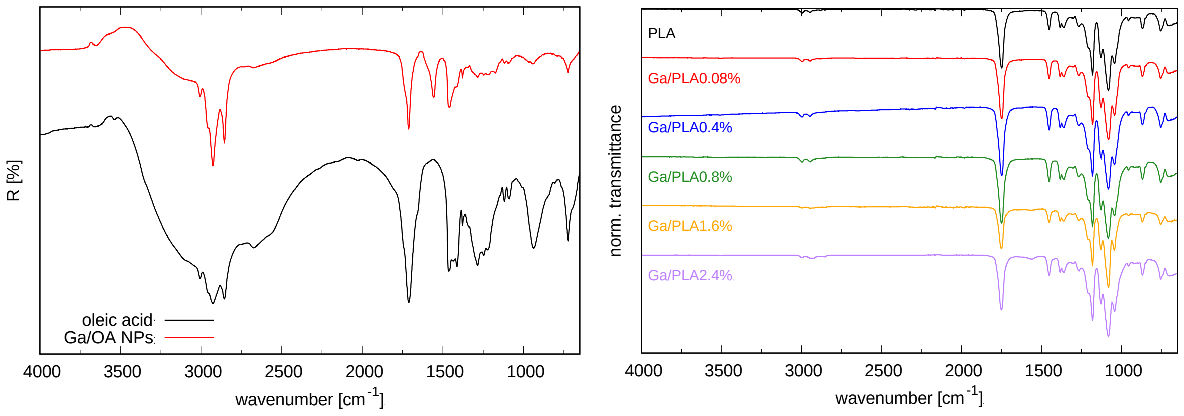

(a) (b)

Figure S2: Additional IR spectra: (a) DRIFT spectrum of OA and colloidal Ga/OA NPs. Both spectra exhibit the most pronounced OA vibrations [5–10]: at 3007 (ν_{as} (=C-H)), 2955 (ν_s (=C-H)), 2927 (ν_{as} (-C-H)), 2855 (ν_s (-C-H), 3000 ((ν(-COO-H, wide), overtones and combinations of δ (OH) (near 1420 cm^{-1}) and ν (C-O) (near 1300 cm^{-1}) in the 2700-2500 cm^{-1} region, 1712 (ν (C=O)), 1465 (δ (CH_2)), 1378 (δ (CH_3)), 1285 (ν (C-O)), 1248 (ν (C-O) dimeric+ ρ_s (*cis*-CH)), 1210+1180 (ν_{as} (C-O-C), doublet), 938 (ω_{in}(O-H··O)) , 723 cm^{-1} (ω_{in}(CH_2)). However, the spectrum of Ga/OA NPs contains an additional band at 1556 cm^{-1}, which is related to COO^- in bidentate bonding of OA to the surface of Ga NPs, while vibrations related to COOH group, including the most prominent C=O vibration at 1712 cm^{-1}, are impeded [9,11–14]. The remaining presence of the band at 1712 cm^{-1} could be due to excess of OA or some OA molecules are also monodentatly bound to the surface of Ga NPs; **(b)** full-range ATR IR spectra of PLA and Ga/PLA nanocomposites in the main article (Fig. 1b).

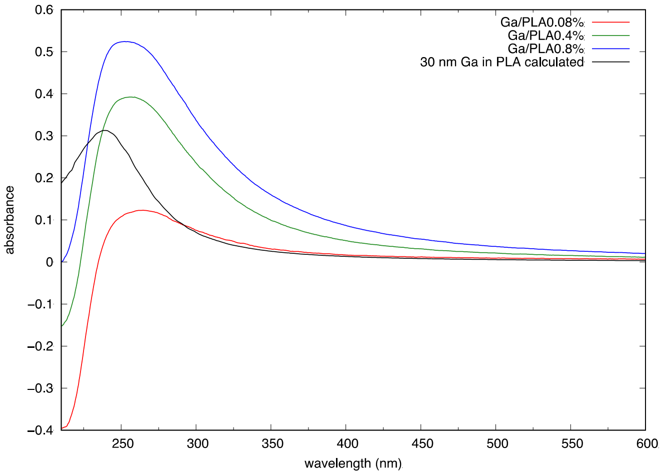

Figure S3: UV-VIS spectra of Ga/PLA after subtraction of spectrum of PLA and calculated UV/VIS spectra of spherical Ga NPs with diameter of 30 nm in PLA (refractive index of 1.52 was taken for calculation).

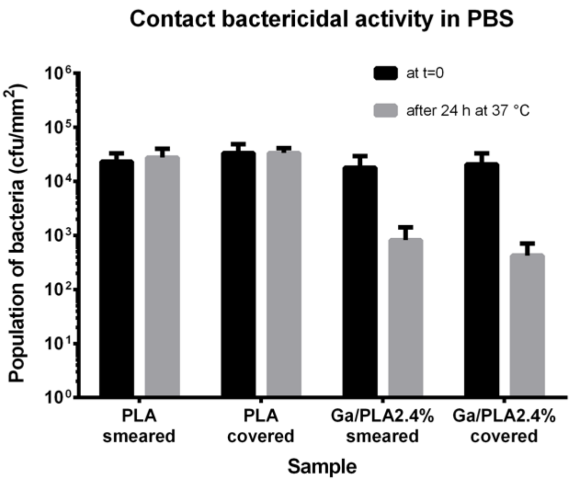

Figure S4: Comparison of two techniques for evaluation of contact bactericidal effect of Ga/PLA in PBS.

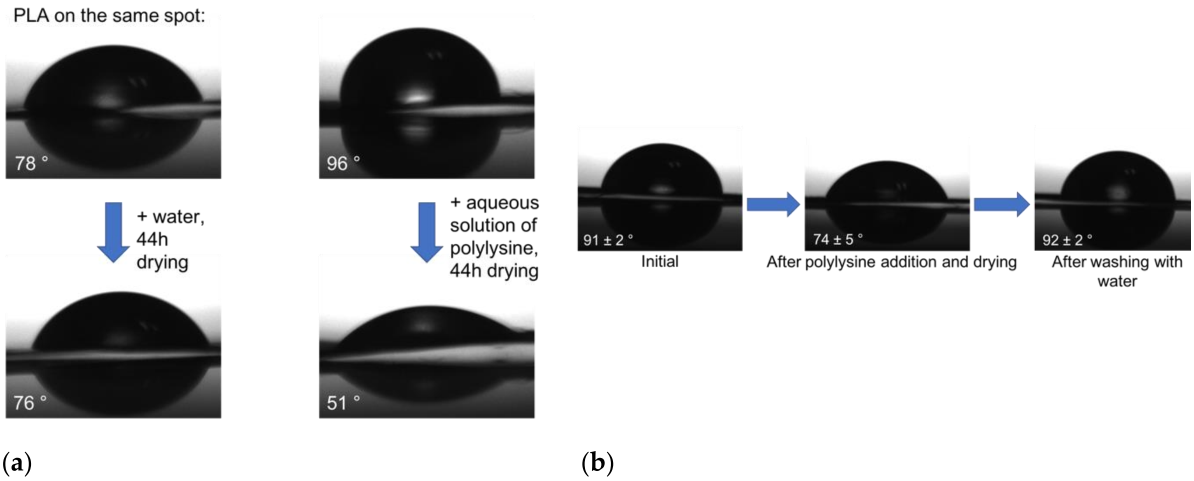

Figure S5. Effect of poly(L-lysine) on the aqueous wettability of the film surface: **(a)** PLA on the same spot, comparison with deionised water as a reference; **(b)** Ga/PLA2.4% average values with representative water droplet images. Washing with water restored the wetting angle that was decreased by polylysine addition.

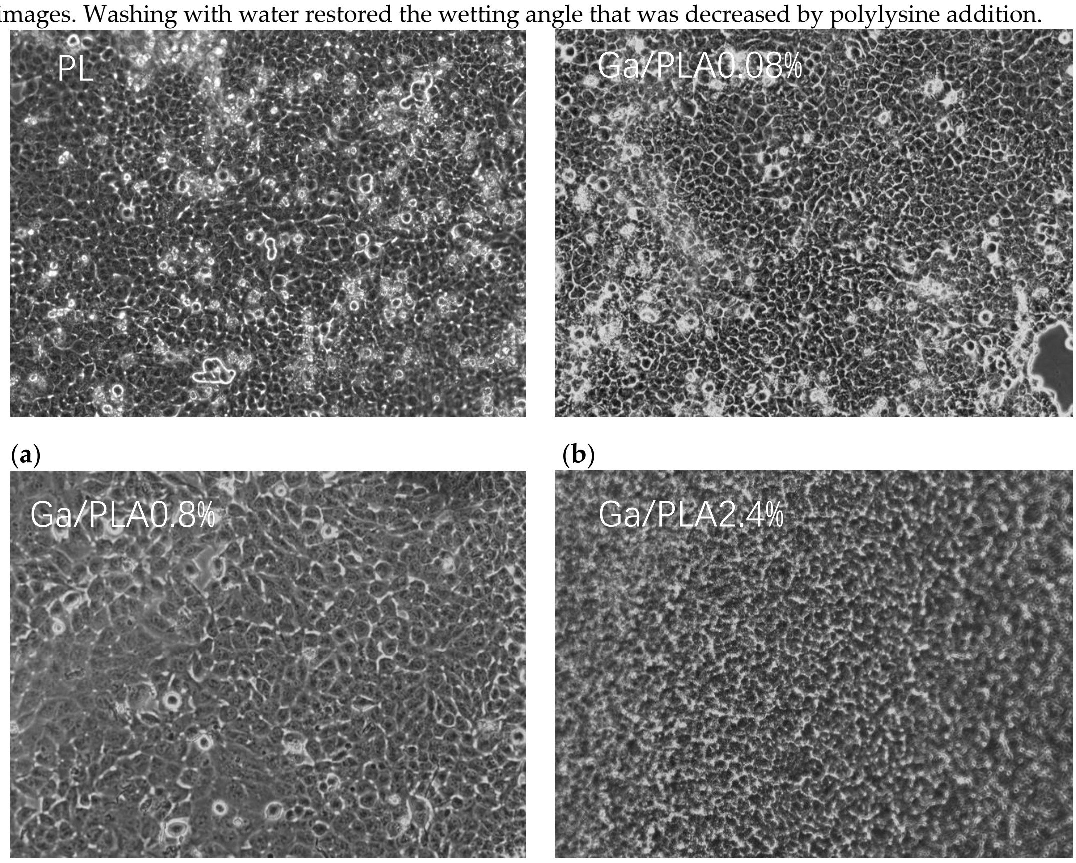

(**a**) (**b**)

(**c**) (**d**)

Figure S6. Phase contrast optical microscope images of HaCaT keratinocytes after three days of growth on the following films: (**a**) PLA; (**b**) Ga/PLA0.08%; (**c**) Ga/PLA0.8%; (**d**) Ga/PLA2.4%.

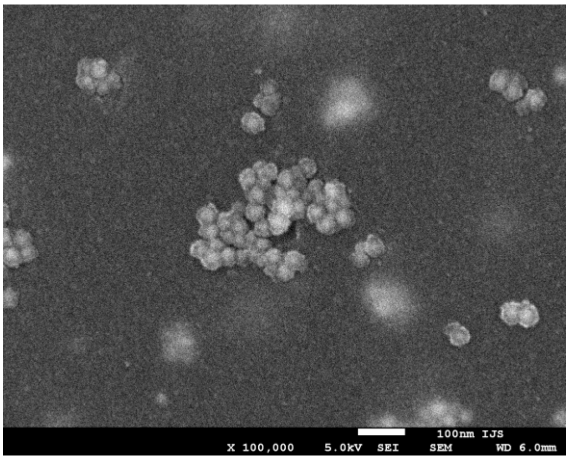

Figure S7. SEM image of non-washed Ga/PLA0.8% after 24-hour exposure to MH liquid.

Supplementary references

1. Mätzler, C. MATLAB Functions for Mie Scattering and Absorption. *IAP Res Rep* **2002**, *2002–08*, 1139–1151.
2. Bohren, C.F.; Huffman, D.R. *Absorption and Scattering of Light by Small Particles*; John Wiley & Sons, Ltd, 1998; ISBN 9780471293408.

3. Knight, M.W.; Coenen, T.; Yang, Y.; Brenny, B.J.M.; Losurdo, M.; Brown, A.S.; Everitt, H.O.; Polman, A. Gallium Plasmonics : Deep Subwavelength Spectroscopic Imaging of Single and Interacting Gallium Nanoparticles. *ACS Nano* **2015**, *9*, 2049–2060.
4. Hutchinson, M.H.; Dorgan, J.R.; Knauss, D.M.; Hait, S.B. Optical properties of polylactides. *J. Polym. Environ.* **2006**, *14*, 119–124, doi:10.1007/s10924-006-0001-z.
5. Silverstein, R.M.; Webster, F.X.; Kiemle, D. *Spectrometric Identification of Organic Compounds*; 7th ed.; Wiley: Hoboken, 2005; ISBN 1118311655.
6. Sinclair, R.G.; McKay, A.F.; Myers, G.S.; Norman Jones, R. The Infrared Absorption Spectra of Unsaturated Fatty Acids and Esters. *J. Am. Chem. Soc.* **1952**, *74*, 2578–2585, doi:10.1021/ja01130a035.
7. Lin-Vien, D.; Colthup, N.B.; Fateley, W.G.; Grasselli, J.G. *The Handbook of Infrared and Raman Characteristic Frequencies of Organic Molecules*; Academic Press: London, 1991; ISBN 0124511600.
8. Thistlethwaite, P.J.; Gee, M.L.; Wilson, D. Diffuse reflectance infrared fourier transform spectroscopic studies of the adsorption of oleate/oleic acid onto zirconia. *Langmuir* **1996**, *12*, 6487–6491, doi:10.1021/la9603939.
9. Shukla, N.; Liu, C.; Jones, P.M.; Weller, D. FTIR study of surfactant bonding to FePt nanoparticles. *J. Magn. Magn. Mater.* **2003**, *266*, 178–184, doi:10.1016/S0304-8853(03)00469-4.
10. Kostevšek, N.; Hudoklin, S.; Kreft, M.E.; Serša, I.; Sepe, A.; Jagličić, Z.; Vidmar, J.; Ščančar, J.; Šturm, S.; Kobe, S.; et al. Magnetic interactions and: In vitro study of biocompatible hydrocaffeic acid-stabilized Fe-Pt clusters as MRI contrast agents. *RSC Adv.* **2018**, *8*, 14694–14704, doi:10.1039/c8ra00047f.
11. Wu, N.; Fu, L.; Su, M.; Aslam, M.; Wong, K.C.; Dravid, V.P. Interaction of Fatty Acid Monolayers with Cobalt Nanoparticles. *Nano Lett.* **2004**, *4*, 383–386.
12. Zhao, S.Y.; Don, K.L.; Chang, W.K.; Hyun, G.C.; Young, H.K.; Young, S.K. Synthesis of magnetic nanoparticles of Fe3O4 and CoFe2O4 and their surface modification by surfactant adsorption. *Bull. Korean Chem. Soc.* **2006**, *27*, 237–242, doi:10.5012/bkcs.2006.27.2.237.
13. Deacon, G.B.; Phillips, R.J. Relationships between the carbon-oxygen stretching frequencies of carboxylato complexes and the type of carboxylate coordination. *Coord. Chem. Rev.* **1980**, *33*, 227–250.
14. Dobson, K.D.; McQuillan, A.J. In situ infrared spectroscopic analysis of the adsorption of aliphatic carboxylic acids to TiO2, ZrO2, Al2O3, and Ta2O5 from aqueous solutions. *Spectrochim. Acta - Part A Mol. Biomol. Spectrosc.* **1999**, *55*, 1395–1405, doi:10.1016/S1386-1425(98)00303-5.
